# Alkyl-Glycerol Rescues Plasmalogen Levels and Pathology of Ether-Phospholipid Deficient Mice

**DOI:** 10.1371/journal.pone.0028539

**Published:** 2011-12-06

**Authors:** Pedro Brites, Ana Sofia Ferreira, Tiago Ferreira da Silva, Vera F. Sousa, Ana R. Malheiro, Marinus Duran, Hans R. Waterham, Myriam Baes, Ronald J. A. Wanders

**Affiliations:** 1 Nerve Regeneration Group, Instituto de Biologia Molecular e Celular (IBMC), Porto, Portugal; 2 Laboratory Genetic Metabolic Diseases, Academic Medical Center, University of Amsterdam, Amsterdam, The Netherlands; 3 ICBAS, Universidade do Porto, Porto, Portugal; 4 Laboratory for Cell Metabolism, Department of Pharmaceutical Sciences, Katholieke Universiteit Leuven, Leuven, Belgium; Governmental Technical Research Centre of Finland, Finland

## Abstract

A deficiency of plasmalogens, caused by impaired peroxisomal metabolism affects normal development and multiple organs in adulthood. Treatment options aimed at restoring plasmalogen levels may be relevant for the therapy of peroxisomal and non-peroxisomal disorders. In this study we determined the in vivo efficacy of an alkyl glycerol (AG), namely, 1-O-octadecyl-rac-glycerol, as a therapeutic agent for defects in plasmalogen synthesis. To achieve this, *Pex7* knockout mice, a mouse model for Rhizomelic Chondrodysplasia Punctata type 1 characterized by the absence of plasmalogens, and WT mice were fed a control diet or a diet containing 2% alkyl-glycerol. Plasmalogen levels were measured in target organs and the biochemical data were correlated with the histological analysis of affected organs. Plasmalogen levels in all peripheral tissues of *Pex7* KO mice fed the AG diet for 2 months normalized to the levels of AG fed WT mice. In nervous tissues of *Pex7* KO mice fed the AG-diet, plasmalogen levels were significantly increased compared to control fed KO mice. Histological analysis of target organs revealed that the AG-diet was able to stop the progression of the pathology in testis, adipose tissue and the Harderian gland. Interestingly, the latter tissues are characterized by the presence of lipid droplets which were absent or reduced in size and number when ether-phospholipids are lacking, but which can be restored with the AAG treatment. Furthermore, nerve conduction in peripheral nerves was improved. When given prior to the occurrence of major pathological changes, the AG-diet prevented or ameliorated the pathology observed in *Pex7* KO mice depending on the degree of plasmalogen restoration. This study provides evidence of the beneficial effects of treating a plasmalogen deficiency with alkyl-glycerol.

## Introduction

Ether-phospholipids are major constituents of cellular membranes and are characterized by an ether-bond at the sn-1 position of the glycerol backbone. Ether-phospholipids are divided into two groups, the distinctive feature being the presence of either a 1-O-alkyl or 1-0-alkenyl side-chain at *sn*-1. Plasmalogens represent the ether-phospholipids with a vinyl-ether linkage (alkenylacyl-glycerophospholipids) whereas platelet-activating factor (PAF) is a typical example of the ether-phospholipids with an 1-O-alkyl ether linkage (alkylacyl-glycerophospholipids) [1], [2]. The biosynthetic pathway of ether-phospholipids, involves several enzymatic steps performed in peroxisomes and the endoplasmatic reticulum [3], [4]. A genetic deficiency affecting either the biogenesis of peroxisomes or one of the two peroxisomal enzymes, i.e. glyceronephosphate O-acyltransferase (Gnpat) and alkylglycerone phosphate synthase (Agps) involved in ether-phospholipid biosynthesis, leads to absent or reduced levels of ether-phospholipids [5]–[8]. In human peroxisomal disorders, the measurement of plasmalogen levels is the hallmark for diagnosis, whereas the levels of PAF have not been fully investigated [9]–[12]. In mammals, the distribution and composition of plasmalogens varies between different tissues with brain, kidney and testes having relatively high levels of plasmalogens.

Plasmalogens contain at the sn-1 position a long chain alcohol composed of either C16:0, C18:0 or C18:1, whereas the sn-2 position contains a polyunsaturated fatty acid (e.g. docosahexaenoic acid, arachidonic acid) [3]. Plasmalogens have been implicated in several biological processes and have been shown to mediate fluidity, signal transduction and to protect against oxidative stress [13], [14]. The human peroxisomal disorder Rhizomelic Chondrodysplasia Punctata (RCDP) type 1 [15], [16], caused by mutations in the *PEX7* gene is characterized by a clinical presentation that includes congenital cataracts, proximal shortening of long bones, contractures and hypotonia [17]–[19]. The deficiency in *PEX7*, which encodes the receptor for peroxisomal proteins containing a peroxisomal targeting signal type 2 (PTS2) impairs the import of three peroxisomal proteins, namely acetyl-CoA acyltransferase 1 (Acaa1), phytanoyl-CoA 2-hydroxylase (Phyh) and Agps [20]–[22]. Despite this triple deficiency, the main pathophysiological factor in RCDP type 1 patients is the severe deficiency in the biosynthesis of plasmalogens, due to the absence of Agps from peroxisomes [23]–[25]. Indeed, strikingly similar clinical features are seen in two other forms of RCDP, namely type 2 and type 3, as caused by mutations in *GNPAT* and *AGPS*, respectively [5], [26]. All types of RCDP share the same clinical presentations which demonstrate that the deficiency in ether-phospholipids is the major cause of tissue pathology and disease state, and highlight the importance of plasmalogens for human health. In RCDP type 1, the absence of Phyh from peroxisomes causes, in addition, a defect in the α-oxidation of phytanic acid [27]. Since both phytanic acid and its precursor (phytol) are solely derived from dietary sources, phytanic acid levels in RCDP type 1 patients may vary depending on the diet and age of diagnosis [15], [28]–[30]. Since high levels of phytanic acid lead to Purkinje cell death, ataxia, retinitis pigmentosa and peripheral neuropathy [31]–[33], the accumulation of phytanic acid in RCDP type 1 patients may worsen tissue pathology and disease progression [34]. The absence of Acaa1 from peroxisomes in RCDP type 1 does not seem to have a generalized metabolic consequence in very-long-chain fatty acid β-oxidation, since levels of VLCFA in plasma and fibroblasts are normal [35]–[37]. Nevertheless, the defect in Acaa1 may be tissue and cell dependent since increased levels of VLCFA have been observed in some blood cells [37].

Alkyl-glycerols (AG) can enter the plasmalogen biosynthetic pathway downstream of the peroxisomal steps and, when added to cultured cells, have been shown to restore plasmalogen levels [38], [39]. AG have been administered to patients with Zellweger syndrome, the most severe peroxisomal disorder in which all metabolic functions of peroxisomes are defective, but clinical improvement was difficult to evaluate since Zellweger patients have a myriad of other metabolic defects that modulate the disease [40], [41].

In this study we evaluated the efficacy of AG in rescuing the biochemical defects and the pathology caused by the deficiency in plasmalogens. We used the *Pex7* knockout (KO) mouse, as it has a complete deficiency in the biosynthesis of ether-phospholipids and displays all the pathological hallmarks of the human disorder [42]. The deficiency in plasmalogens characteristic of *Pex7* KO mice is due to the impaired import of AGPS into peroxisomes. Similarly to RCDP type 1, *Pex7* KO mice also have an impairment in the import of Phyh and Acaa1. Despite this triad of import deficiencies, the phenotype of *Pex7* KO is primarily, if not solely, due to the defect in plasmalogens since it closely resembles that of the Gnpat KO mouse [43]. Moreover, under standard dietary regiments the *Pex7* KO mice do not accumulate phytanic acid and the accumulation of VLCFA is age and tissue dependent [42], [44].

In this study we evaluated the efficacy of AG in rescuing the biochemical defect and the pathology caused by a deficiency of plasmalogens. We fed WT and *Pex7* KO mice a diet containing the AG, i.e. 1-*O*-octadecyl-rac-glycerol, and found that compared to the control diet, the AG diet increased plasmalogen levels in *Pex7* KO mice and therefore can be used *in vivo* to restore plasmalogen levels. Moreover, the restoration of plasmalogen levels could halt or at least slow down the progression of the pathology in several target organs depending on the pathological status of the target tissues at the time of the therapeutic intervention. Our results demonstrate the benefits/effects of AG as a therapeutic agent in diseases in which there is a defect in ether-phospholipid biosynthesis.

## Results

### Alkyl-glycerol supplementation to *Pex7* KO mice rescues plasmalogen deficiency

To determine whether AG could restore the plasmalogen deficiency present in *Pex7* KO mice, we fed mice either a control diet or a diet containing 2% 1-*O*-octadecyl-rac-glycerol (AG diet) for 2 months. In *Pex7* KO mice fed the AG diet, plasmalogen levels increased from undetectable to the levels found in control-fed or AG-fed wild type mice in erythrocytes and in several tissues including kidney, heart and eye (Table 1). These results indicate that in these tissues the AG diet could restore plasmalogens to the physiological steady-state level. In contrast, the AG diet only marginally increased plasmalogen levels in nervous tissues. In the peripheral nervous system (PNS), measurement of plasmalogens in sciatic nerves revealed that in *Pex7* KO fed the AG diet plasmalogen levels increased to 2.3% of WT levels (Table 1). In the central nervous system (CNS), measurement of plasmalogens in cerebrum and cerebellum revealed marginal increases to 0.7% and 1.4% of WT level, respectively, whereas in spinal cord no increase was observed. In order to test if longer treatment periods could increase the plasmalogen levels in CNS tissues we fed *Pex7* KO mice the AG diet for 4 months (Table 1). After this prolonged treatment, plasmalogen levels in spinal cord and cerebellum of treated *Pex7* KO mice were higher than after the 2 month treatment period, displaying 2.9% of WT levels. These results indicate that AG can be used to increase plasmalogen levels *in vivo* and that different treatment periods should be considered depending if the target is systemic or nervous tissue.

**Table 1:** Plasmalogen levels in different tissues after control and AG-diet feeding.

		Control diet		AG diet	
Period	Sample	WT	KO	WT	KO
2 months	RBC	4.2±0.2	0.1±0.1	7.6±0.7	5.4±0.3
	Liver	0.5±0.1	N.D.	1.1±0.1	1.4±0.1
	Kidney	3.5±0.2	N.D.	7.1±0.5	3.9±0.2
	Heart	2.3±0.4	N.D.	9.8±1.6	9.6±1.1
	Lung	8.1±0.9	N.D.	15.7±2.1	17.7±3.2
	Testis	2.4±0.1	N.D.	3.4±0.2	2.1±0.5
	Eye	4.0±0.1	N.D.	3.1±0.6	2.7±0.2
	Sciatic nerve	26.7±7.1	N.D.	39±1.5	0.6±0.12
	Cerebrum	13.7±2.8	N.D.	13.9±1.9	0.1±0.1
	Cerebellum	14.5±1.0	N.D.	14.5 ±0.5	0.2±0.1
	Spinal cord	21.3±6.1	N.D.	20.1±5.1	N.D.
4 months	Cerebellum	N.P.	N.P.	13.7±0.8	0.4±0.06
	Spinal cord	N.P.	N.P.	17.2±2.8	0.5±0.1

Plasmalogen levels are expressed as mean ± S.D of the percentages of dimethylacetal (DMA) derivatives of C18:0 to the corresponding saturated fatty acid.

Numbers of mice tested: on control diet WT mice (n = 4) and *Pex7* KO mice (n = 3) and on AG diet for 2 months WT mice (n = 6) and *Pex7* KO mice (n = 6); for 4 months WT (n = 3) and *Pex7* KO mice (n = 3). RBC- red blood cells; AG- alkyl-glycerol diet; N.D.- not detected; N.P.- not performed.

### Alkyl-glycerol supplementation to *Pex7* KO mice improves the pathology of affected tissues

**Figure 1:**
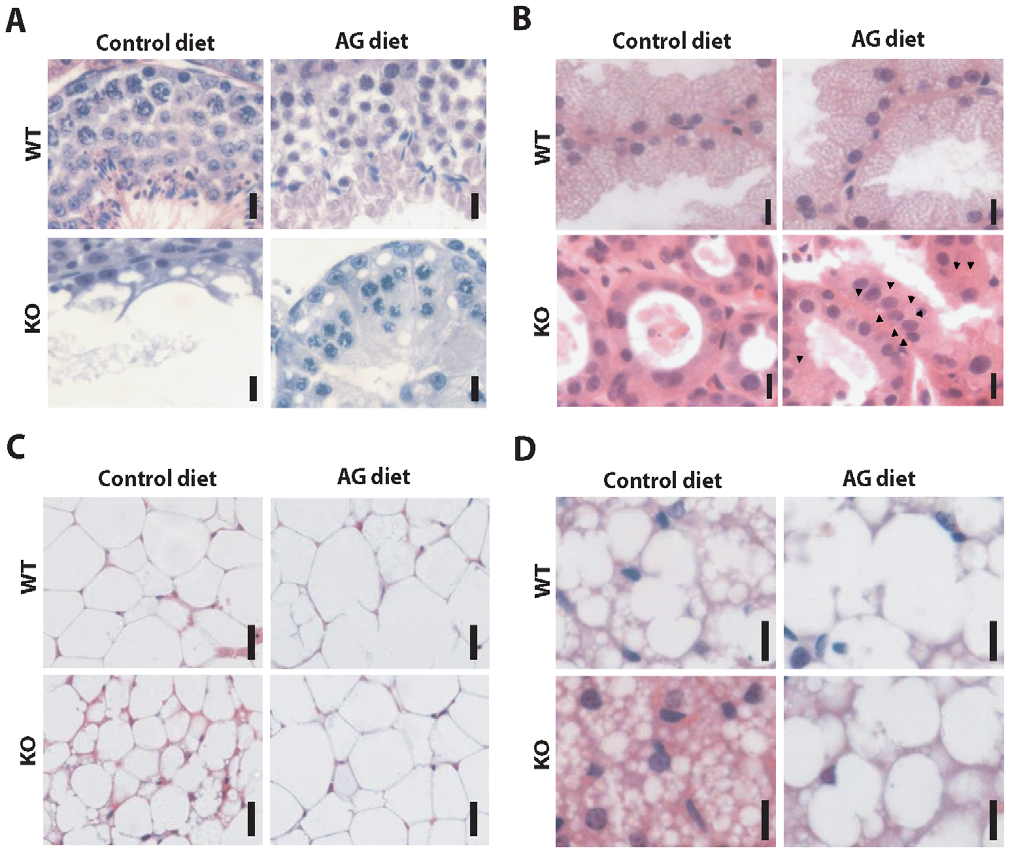
Therapeutic effects of AG diet on rescuing the pathology caused by plasmalogen deficiency. (A) Testis sections stained with hematoxylin and eosin (H&E). Seminiferous tubules from control-fed *Pex7* KO mice have no spermatocytes and display a Sertoli-only phenotype, whereas seminiferous tubules from AG-fed *Pex7* KO mice display a stratified epithelium with spermatocytes at different stages of maturation. Bars are 10 µm. (B) Harderian gland sections stained with H&E. After AG diet, Harderian glands from *Pex7* KO mice showed a restoration in morphology that included the appearance of small lipid inclusions (arrowheads) and increased size of glandular cells. Bars are 10 µm. (C) White adipose tissue sections stained with H&E. Degenerated adipocytes with small-sized fat inclusions are characteristic features found in *Pex7* KO mice. After AG diet, adipocytes from *Pex7* KO mice displayed normal size and fat content. Bars are 25 µm. (D) Brown adipose tissue sections stained with H&E. Adipocytes from control-fed *Pex7* KO mice showed, in contrast to WT, an increased number of small fat inclusions within the cytoplasm of adipose cells. AG-diet restored the histology of adipocytes in brown adipose tissue of *Pex7* KO mice. Bars are 10 µm. Numbers of mice analyzed from two independent cohorts: on control diet WT mice n = 4 and *Pex7* KO mice n = 3, on AG diet WT mice n = 6 and *Pex7* KO mice n = 6.

Next, we determined the effects of the AG diet on the pathology observed in *Pex7* KO mice. Testicular atrophy primarily caused by loss of cells from the spermatogonia lineage is one of the hallmarks of the pathology caused by deficiencies in the biosynthesis of plasmalogens in mice [44], [45]. Histological evaluation of seminiferous tubules from *Pex7* KO mice on control diet showed a Sertoli-only phenotype, with the seminiferous epithelium solely populated by Sertoli cells and devoid of spermatogonia and spermatocytes (Fig. 1A). Treatment of *Pex7* KO mice with the AG diet for 2 months restored plasmalogen levels in the testis and ameliorated the testicular pathology as evident from the presence of spermatocytes at different stages of maturation in the seminiferous epithelium (Fig. 1A), which is indicative of a restoration in spermatogenesis. Regardless of the histological improvement, however, mature spermatozoa were not detected in the seminiferous tubules or in the epididymis (data now shown).

**Figure 2:**
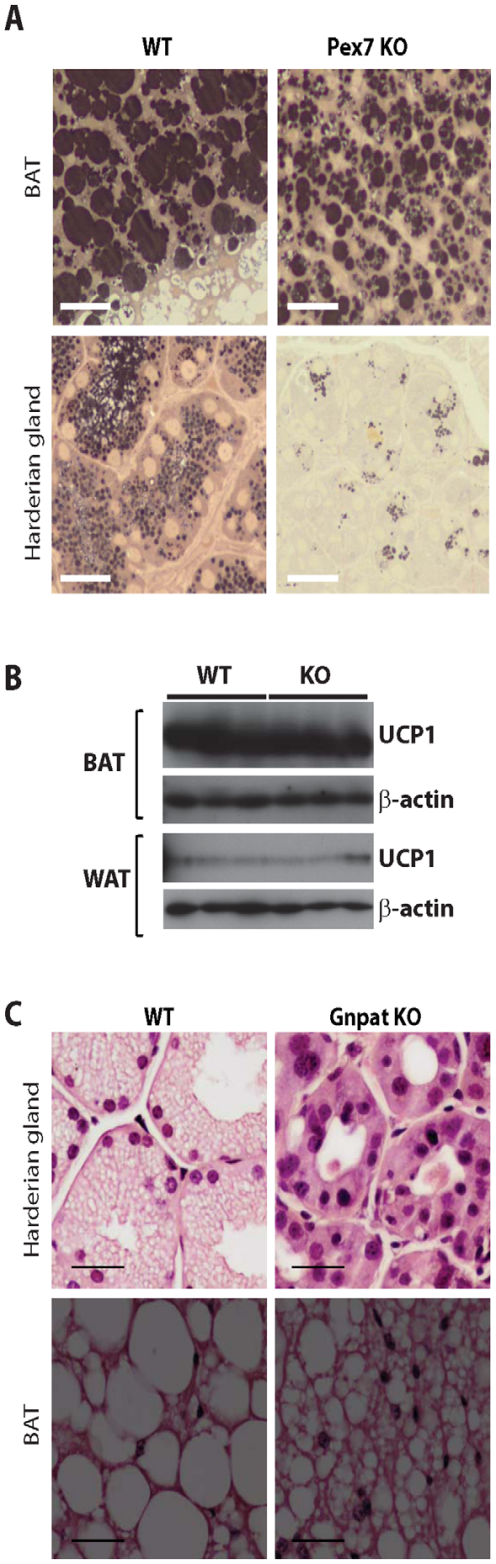
Lipid rich tissues are affected by a deficiency in plasmalogens. (A) Lipid staining of BAT (upper panels) and Harderian glands (bottom panels) from 3 months old WT and Pex7 KO mice. Adipocytes from Pex7 KO mice, showed portioning of the lipid inclusions with smaller lipid droplets. The secretory cells in the Harderian glands from Pex7 KO mice were characterized by an extremely reduced content of lipid inclusions. Bars are 25 µm. (B) Western blot analyses of UCP1 expression in BAT and WAT lysates from 3 months old WT and Pex7 KO mice. Analysis of β-action was used as loading control. In BAT the characteristic high levels of UCP1 expression did not differ in Pex7 KO samples. In WAT, the considerable reduced expression of UCP1 was not altered in lysates from Pex7 KO mice, indicating no major disturbances in lypolysis that could explain the altered histological appearance of BAT and WAT. (C) Histological assessment with hematoxylin and eosin (H&E) staining of Harderian gland and BAT from 3 months old WT and Gnpat KO mice. Gnpat KO mice have a defect in the biosynthesis of plasmalogens, and also display the loss of lipid inclusions in Harderian glands (upper panels) and abnormal brown adipocytes (bottom panels), with irregular and smaller lipid droplets. Bars are 25 µm.

The Harderian gland synthesizes lipids, porphyrins and indoles for pheromonal and lubricatory purposes, and has been implicated in, amongst others, thermoregulatory and photoreceptor protection processes [46]. The lipids which are enriched in alkylglycerols are stored in numerous small droplets and are subsequently excreted (Fig. 1B). Harderian glands contain peroxisomes [47], [48] and show gross abnormalities in case of peroxisomal dysfunction (Brites et. al. unpublished results, Fig. 1B and Fig. 2A). Histological examination of Harderian glands from *Pex7* KO mice showed atrophic secretory cells with reduced cytoplasm, lacking the characteristic lipid inclusions found in Harderian glands of WT mice (Fig. 1B and Fig. 2A). Treatment of *Pex7* KO mice with the AG diet for 2 months improved the histology of the Harderian gland with secretory cells showing small lipid droplets and an increase in the cytoplasmic volume (Fig. 1B).

It is quite striking that also adipose tissue, which is the most important storage of lipids in the body, is abnormal in plasmalogen deficient mice [44]. Epididymal, inguinal, retroperitoneal and subscapular white adipose tissue (WAT) depots are extremely reduced, whereas the sizes of the dorsal brown fat pads are normal. Histological analyses of lipids in brown adipose tissue (BAT) from WT and Pex7 KO mice (Fig. 1D and Fig. 2A) revealed abnormally small lipid droplets within the cytoplasm of individual adipocytes. The extremely reduced WAT and abnormal histology of BAT was not due to a reduced food intake (WT 5.8±0.5 g/day; Pex7 KO 4.9±0.8 g/day; non-significant difference with p = 0.1). Analysis by western blot of uncoupling protein 1 (UCP1) in BAT and WAT of WT and Pex7 KO mice, did not reveal any differences (Fig. 2B), suggesting that in Pex7 KO mice there is no deregulation of uncoupling activity that would explain the loss of WAT and the lipolysis-like phenotype of BAT.

To obtain further evidence that lipid droplet abnormalities are caused by ether-phospholipid deficiency, we performed a histological assessment in the Harderian gland and BAT of Gnpat KO mice and WT littermates (Fig. 2C). Gnpat KO mice are characterized by a unique defect in the biosynthesis of plasmalogens [43]. The histological analyses showed that the Harderian gland of mutant mice is devoid of the characteristic lipid inclusions, similar to *Pex7* KO mice. In addition, BAT from Gnpat KO mice also showed partitioned fat deposits and abnormally small lipid droplets. Since both mutant mice with defects in the biosynthesis of plasmalogens share the same lipid abnormalities in Harderian glands and adipose tissues, our results suggest a role of plasmalogens in lipid droplet formation and/or maintenance.

Based on these findings, adipose tissue was also used to evaluate the efficacy of the AG diet in *Pex7* KO mice. The AG diet normalized the histological appearance of white adipocyte size and fat deposition in *Pex7* KO mice (Fig. 1C), and also produced beneficial effects on brown adipocytes, characterized by the increased size of lipid inclusions (Fig. 1D). This was accompanied by a significant increase in body weight of Pex7 KO mice. Indeed, on a normal diet the mutant mice weighed considerably less that WT mice (22.0 gr ±1.1 (n = 3) vs. 33.3 gr ±1.5 (n = 4) p = 0.0014). Feeding the AG diet for 2 months led to an increase in body weight of *Pex7* KO mice (KO 28.7 gr ±1.7 (n = 6); WT 32.3 gr ±1.8 (n = 6)) representing a 30% gain (p = 0.0048) when compared to untreated *Pex7* KO mice.

**Figure 3:**
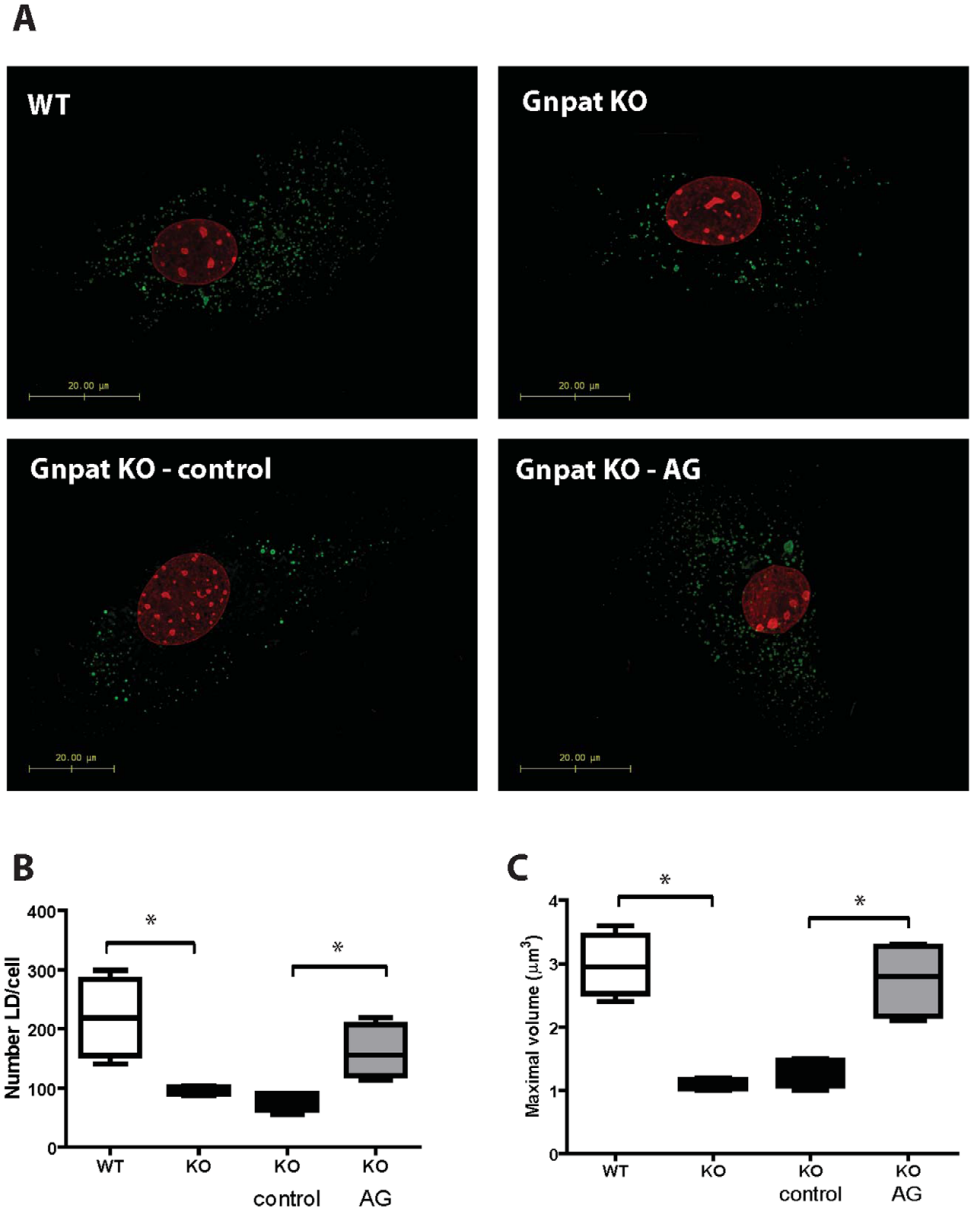
Deficiency in plasmalogens affects lipid droplet formation in MEFs. (A) Lipid droplets were stained with BODIPY 493/503 in MEFs from WT and Gnpat KO mice (n = 4 per genotype). MEFs were cultured in normal medium (upper panels), in control medium (bottom left panel) or in medium supplemented with 15 µM AG (bottom right panel). In MEFs from Gnpat KO mice, the deficiency in plasmalogens leads to a reduction in the number and volume of lipid droplets (B, C). Treated of Gnpat KO MEFs with 15 µM AG for 7 days lead to an increase in the number and volume of lipid droplets (B, C) to values similar to those of lipid droplets from WT MEFs. * p<0.02 using Mann Whitney test.

Taken together, the results obtained highlight the importance of plasmalogens in the accumulation of lipid inclusions in Harderian glands and adipose tissue. To further evaluate the role of plasmalogens in lipid droplet formation, we prepared mouse embryonic fibroblasts (MEFs) from WT and Gnpat KO E13.5 embryos. Analysis of lipid droplets under normal culture conditions showed that, plasmalogen-deficient MEFs had fewer and smaller lipid droplets when compared to WT MEFs (Fig.3A–C). The *in vitro* treatment of Gnpat KO MEFs with 15 µM AG, led to a restoration of the number and volume of lipid droplets (Fig. 3A–C). These results indicate that plasmalogens are important for lipid droplet homeostasis, and that treatment with AG can rescue the defects in lipid droplet formation.

**Figure 4:**
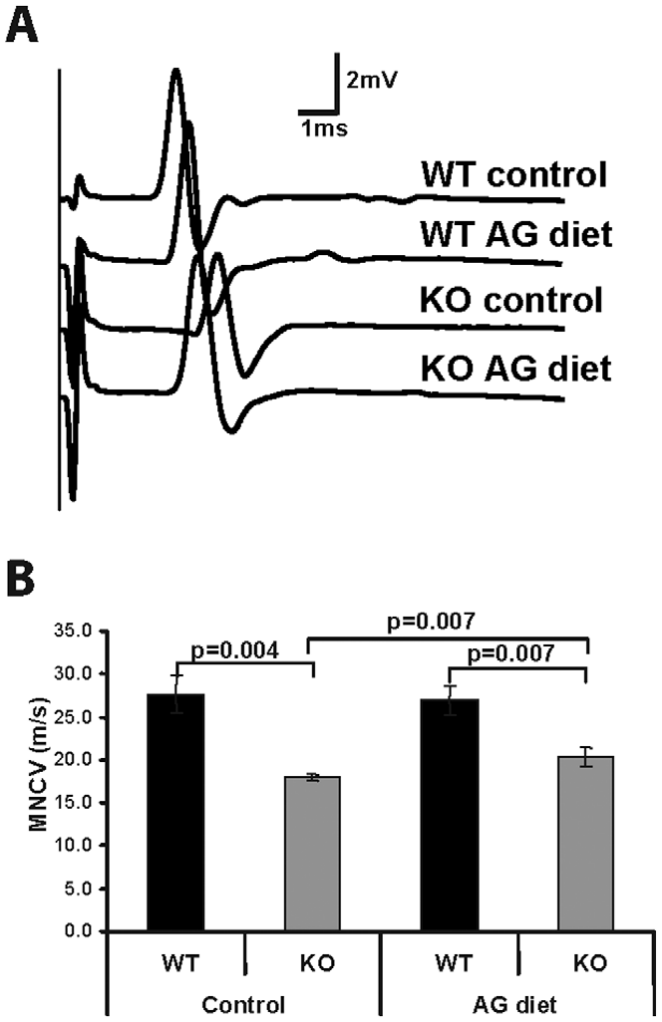
Nerve conduction improves after AG treatment. (A) Representative examples of compound muscle action potentials recordings after stimulation at the sciatic notch of wild type and *Pex7* KO mice fed control or AG diets. Increased latencies were observed in control-fed *Pex7* KO mice that were partially restored after the AG diet. (F) Calculated motor nerve conduction velocities (MNCV) of wild type and *Pex7* KO mice fed control or AG diets. Bars represent the average values obtained after bilateral measurements in wild type and *Pex7* KO mice, and the significance is shown above each comparison. The AG diet partially restores MNCV in sciatic nerves of *Pex7* KO mice. Numbers of mice analyzed: on control diet WT mice n = 4 and *Pex7* KO mice n = 3, on AG diet WT mice n = 4 and *Pex7* KO mice n = 4.

We also evaluated the effects of the AG diet on the functioning of peripheral nerves since we observed an increase in plasmalogen levels after the AG diet. We found that *Pex7* KO mice develop a peripheral neuropathy with reduced motor nerve conductance velocity (MNCV). On the control diet, *Pex7* KO mice showed increased latencies of compound muscle action potentials (CMAPs) (Fig. 4A) that resulted in a 37% reduction in MNCV when compared to WT mice on the control diet (Fig. 4B). *Pex7* KO mice on the AG diet showed an improvement in CMAP latencies (Fig. 4A) that resulted in an increase in MNCV. Although the AG diet did not restore the MNCV of *Pex7* KO mice to normal values, it clearly improved nerve conduction, with KO mice having only a 19% reduction in MNCV when compared to WT mice (Fig. 4B).

These results indicate that the increase in plasmalogen levels observed after the treatment with the AG diet can ameliorate the pathology in testis and Harderian gland. Treatment with the AG diet also restored adipose tissue morphology and let to improved nerve function of *Pex7* KO mice. In addition, the observed defect in lipid droplets of MEFs from Gnpat KO mice could also be reverted with the *in vitro* AG-treatment.

### Alkyl-glycerol treatment before onset of pathology

Our combined results indicate that the AG diet had dual beneficial effects since it not only restores plasmalogen levels but it also improved the histopathological alterations in target organs of *Pex7* KO mice. This led us to investigate if the AG diet could also be used to prevent the pathology caused by plasmalogen deficiency. To achieve this, we fed the control or AG diet to pregnant dams and determined the effects on 20-day old pups (P20). Regardless of the diet fed to the dams, *Pex7* KO pups were born hypotonic and measurements of body weight during the first 2 postnatal weeks showed a significant difference when compared to WT pups (20% reduction in body weight; p = 0.005), which suggests that AG did not cross the placental barrier and/or was not present in milk during lactation. Nevertheless, after the second postnatal week the weight of *Pex7* KO pups from the AG diet increased to reach that of WT pups (with KO mice having only 5 to 12.5% reduction in body weights when compared to WT mice; p = 0.726). Combined with the previous results of increased body weight gain from the dietary regimen on adult mice, these results suggest that the increase in body weight of *Pex7* KO pups starts upon the oral ingestion of the AG diet.

**Figure 5:**
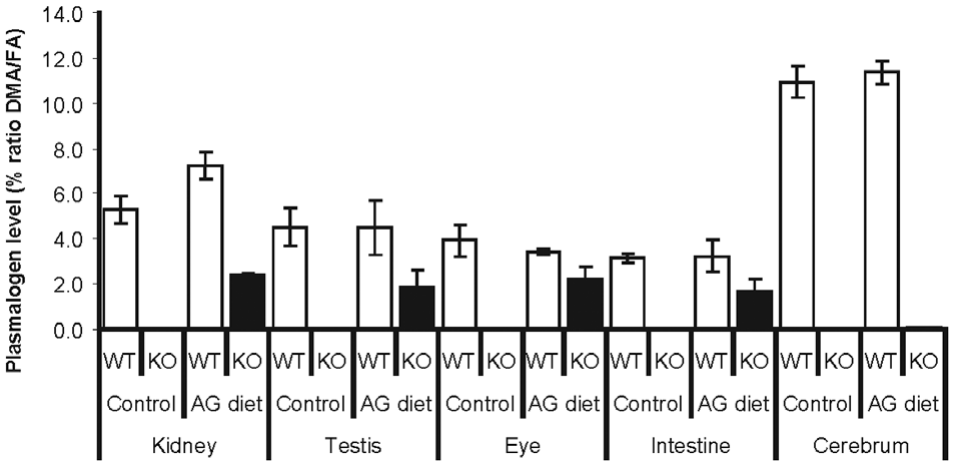
Plasmalogen levels after AG treatment in young mice. Plasmalogen levels in several tissues from 20-days old (P20) mice fed a control diet (WT pups n = 4; *Pex7* KO pups n = 4) or an AG diet (WT pups n = 5; *Pex7* KO pups n = 6). Increased levels of plasmalogens are observed in kidney, testis, eye and intestine.

Measurement of plasmalogen levels showed that the AG diet led to increased plasmalogen levels in multiple tissues of *Pex7* KO pups (Fig. 5). The restoration of plasmalogen levels in systemic tissues from *Pex7* KO pups varied between 45 and 65% of the WT levels. This partial restoration of plasmalogens was likely due to the source of AG in *Pex7* KO pups which was primarily diet-derived and the pups ingested the AG diet for a period of maximally 6 days. The undetectable levels of plasmalogens in cerebrum of AG-treated *Pex7* KO mice are likely due to the short period of treatment, since the increases in plasmalogens observed in older mice required a treatment for 2 or 4 months (Table 1).

**Figure 6:**
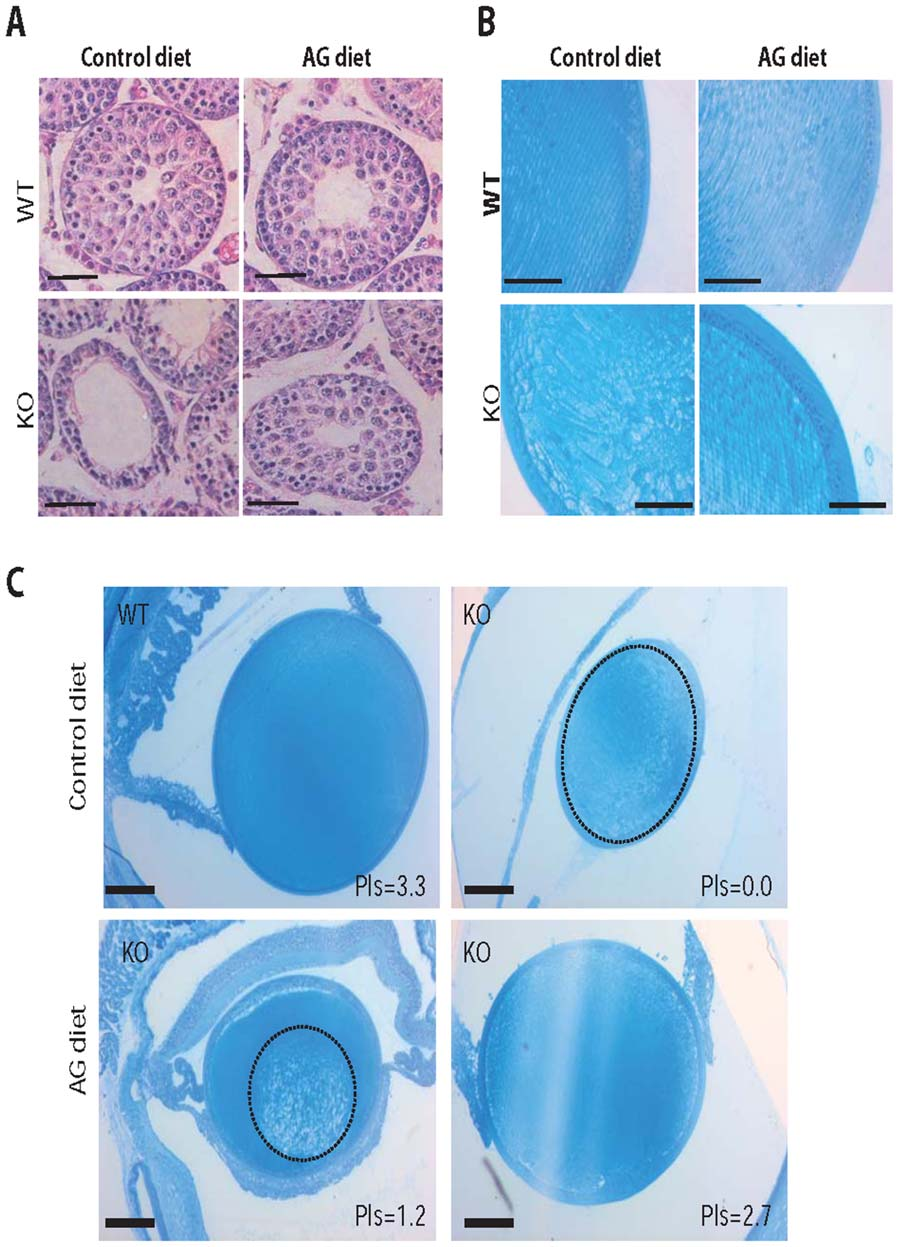
AG treatment in young mice prevents tissue pathology. (**A**) Testis sections of P20 WT and *Pex7* KO pups stained with hematoxylin and eosin (H&E). Whereas control-fed *Pex7* KO pups already showed the degenerative changes in spermatocytes, AG-fed *Pex7* KO pups where protected from degeneration and showed normal appearing seminiferous tubules and spermatocytes. Bars are 50 µm. (B) Eye sections of P20 WT and *Pex7* KO pups stained with Richardson's stain. In contrast to the lens of WT pups showing organized and orientated fiber cells, the cataract in the lens of control-fed *Pex7* KO pups shows abnormally sized and abnormally arranged fiber cells. The AG diet prevented the abnormal development of fiber cells in *Pex7* KO pups thus preventing cataract formation. Bars are 50 µm. (C) Eye sections of mice showing the correlation between plasmalogen (Pls) levels and the development of cataracts in *Pex7* KO pups (from each mouse one eye was used for histology and the other eye used for biochemical analyses). Whereas complete loss of plasmalogens (Pls = 0.0) leads to a massive cataract occupying the entire lens (the cataract area is circled with a dashed line), a plasmalogen level of 2.7% is able to prevent cataract formation in *Pex7* KO pups. Partial restoration of plasmalogens (Pls = 1.2%) leads a small nuclear cataract (circled with a dashed line). Sections were stained as in (B) and bars are 100 µm. Numbers of mice analyzed: on control diet WT mice n = 4 and *Pex7* KO mice n = 4, on AG diet WT mice n = 4 and *Pex7* KO mice n = 6.

Next, we determined the effects of the AG diet on the histopathology of *Pex7* KO pups. At P20, testis of *Pex7* KO pups fed the control diet showed disorganization of the seminiferous epithelium with loss of spermatocytes (Fig. 6A), whereas *Pex7* KO pups fed the AG diet showed normal seminiferous tubules without loss of spermatocytes (Fig. 6A) indicating that the 42% restoration in plasmalogen levels (Fig. 5) could prevent testicular degeneration.

The AG diet also prevented the development of cataracts in *Pex7* KO pups. Whereas *Pex7* KO pups fed a control diet showed bilateral cataracts as soon as the pups opened the eye lids (age P14-P15), AG-fed *Pex7* KO pups failed to develop cataracts or developed a unilateral small nuclear cataract (Fig. 6B). The presence and the extent of the cataract in AG-fed *Pex7* KO pups correlated with the amount of plasmalogens measured in the eye (Fig. 6C).

These results indicate that an early increase in plasmalogen levels can prevent the development and progression of tissue pathology.

## Discussion

As highlighted by the clinical presentation of RCDP patients, ether-phospholipid deficiency has major consequences for tissue development and organ function [19]. Therefore any therapeutic intervention aimed at restoring ether-phospholipid levels should have a beneficial effect. In recent years deficiencies in plasmalogen levels have been observed in other peroxisomal diseases as well as non-peroxisomal disorders [49]–[52], which increases the need for the development and implementation of a method to increase plasmalogen levels.

*In vitro* studies have shown that alkyl-glycerols are able to enter the plasmalogen biosynthetic pathway after the initial peroxisomal steps, and restore plasmalogen levels [38], [53], [54]. AG also have other beneficial advantages that include reduced side effects of radiotherapy, inhibition of tumor growth, and stimulation of the immune system [55]. Nevertheless, their *in vivo* application as precursors of plasmalogens has remained limited [40], [56], [57]. Using the *Pex7* KO mouse as a model for RCDP type 1 and as a model characterized by a complete deficiency of ether-phospholipids, we have evaluated the *in vivo* effectiveness of AG as a plasmalogen precursor and as a therapeutic agent. Treatment of WT and *Pex7* KO mice with the AG 1-*O*-octadecyl-rac-glycerol led to the restoration of plasmalogens containing a C18:0 moiety at the sn-1 position of the glycerol backbone. The most abundant forms of plasmalogens are those containing either a C16:0 or a C18:0 at the sn-1 position. Using 1-*O*-octadecyl-rac-glycerol as a precursor we rescued the plasmalogen form containing the C18:0 moeity. The importance or functionality of the different forms of plasmalogens is not well established, but our work suggests that the combination of different AG, e.g. a mixture of 1-*O*-octadecyl-rac-glycerol with 1-O-hexadecyl-rac-glycerol, would be able to rescue the two most abundant forms of plasmalogens containing C18:0 and C16:0 moieties, respectively. In our setup we used a diet supplemented with 2% w/w AG, which translates to a daily dosage of 80mg AG per mouse per day. The diet was well tolerated as judged by visual inspection of mice and the biochemical, histological and functional assessments performed. Recently, Braverman et al., reported the generation of a hypomorphic *Pex7* mouse, and evaluated the ability of the AG 1-*O*-octadecyl-rac-glycerol to rescue plasmalogen levels in erythrocytes [57]. In contrast to the *Pex7* KO mouse, in which plasmalogens levels are undetectable, the hypomorphic *Pex7* model shows on average a 50-60% decrease in plasmalogen levels. The AG diet (1mg AG per mouse per day) increased but did not normalize plasmalogen levels in erythrocytes of hypomorphic *Pex7* mice [57]. This is possibly due to the low dosage of AG used. In addition, 1-*O*-octadecyl-rac-glycerol can undergo oxidative cleavage of its alkyl bond by the action of glyceryl-ether monooxygenase [58], which can decrease the availability of AG for the biosynthesis of plasmalogens. In our study, the use of a higher dosage of AG, may circumvent the loss of 1-*O*-octadecyl-rac-glycerol through the actions of glyceryl-ether monooxygenase and/or lysophospholipases. In humans well tolerated dosages of AG range from 5–50 mg/kg [40], and the dietary supplements of shark liver oils may contain up to 200 mg of AG.

In systemic tissues of adult *Pex7* KO mice, the AG diet was able to restore plasmalogen levels to physiologic levels, showing its efficacy as a plasmalogen precursor. Nevertheless, the inefficient rescue of plasmalogen levels in nervous tissue by the AG diet remains to be addressed. In the CNS, the blood-brain barrier and/or the increased catabolism of newly formed plasmalogens, with half-lives of less than 1 hour [59], as well as the metabolic heterogeneity found within different areas of the brain towards plasmalogen biosynthesis may explain the inability of the AG diet to normalize plasmalogen levels [40], [59], [60]. Moreover, since nervous tissues is highly enriched in plasmalogens, longer treatment periods may be necessary to overcome the turnover rates and to reach steady-state physiologic levels. In the PNS we observed an improvement of the neuropathic condition in *Pex7* KO mice treated with the AG diet, despite marginal increases in plasmalogens. Therefore, longer periods of treatment may have beneficial effects for nervous tissue regardless of the level of plasmalogens restoration.

In adult *Pex7* KO mice, despite a considerable pathology in target tissues (e.g. testis and adipose tissue) the treatment with AG was able to halt the progression of the disease and allow tissue regeneration. An exception was the eye, in which the destruction of the lens fibers and formation of a cataract prior to the start of the treatment could not be reverted by the AG diet (data not shown). In young *Pex7* KO pups, the partial restoration of plasmalogen levels in systemic tissues was most likely due to the inability of AG to cross the placenta barrier and to be secreted in milk [40]. Consequently, the pups ingested the AG diet for a period of only six days. Nevertheless, these results also demonstrate that within a week of treatment, most systemic tissues have reached approximately 50% of WT levels. The study of the major affected tissues in these young pups (i.e., eye and testis), revealed that the AG diet could prevent the testicular degeneration and the cataract formation. Of interest was the correlation between plasmalogen levels and cataract formation, suggesting that at least half the normal levels of plasmalogen are required to prevent the development of cataracts. In RCDP patients, similar results were obtained from the study of patients with mild and severe forms. Patients with milder forms lacking the rhizomelia and cataracts, usually have plasmalogen levels that amount to approximately 35–45% of control levels [28].

In summary, our findings indicate that the *Pex7* KO mouse is an appropriate model to test and evaluate agents aimed at restoring ether-phospholipids. Moreover, our results showed that the AG, 1-*O*-octadecyl-rac-glycerol, can be used *in vivo* to restore plasmalogen levels in peripheral organs and to halt or prevent pathological alterations caused by plasmalogen deficiency. Our results *in vitro* also showed that AG can be used to rescue the lipid droplet defects caused by the deficiency in plasmalogens. Our data raises the exciting prospect of using AG as therapeutic agents making use of its ability to correct plasmalogen levels in disorders caused or modulated by a deficiency in plasmalogens, which ranges from the genetic defects in etherphospholipid biosynthesis to Alzheimer's disease. In addition, our work should also drive the design and evaluation of synthetic plasmalogens [61] or other plasmalogen precursors in order to restore ether-phospholipid levels in disorders where a deficiency in ether-phospholipids causes or modulates the disease state.

## Materials and Methods

### Animals

*Pex7* KO mice and littermate WT mice in a Swiss Webster background were obtained by mating *Pex7* heterozygous mice and genotyped as described previously [42]. Gnpat KO mice [43] were kindly provided by Prof. W. Just (Heidelberg, Germany) and have been backcrossed to Swiss Webster for 7 generations. Mice were housed under standard conditions and had free access to food and water. For tissue harvesting, mice were anesthetized with 100mg/kg ketamine and 10mg/kg xylazine. Blood was collected by cardiac puncture and isolated organs were snap-frozen in liquid nitrogen and stored at −80°C for further analyses. To determine food consumption, WT and Pex7 KO mice (n = 5 for each genotype) were individually housed and the weight of the food pellets was measured every morning during a period of 10 days. Experiments and mouse manipulations were approved by the Animals Experiments Committee of the University of Amsterdam (GMZ11 ID100351 and GMZ1011 ID100924) and by the Direção Geral de Veterinária (DGV; project 011852).

### Diet study

Alkyl-glycerol supplemented diets containing 2% 1-*O*-octadecyl-rac-glycerol (Sigma-Aldrich) dissolved in ethanol and the control diets lacking alkyl-glycerol were either manufactured by Ab Diets (Woerden, the Netherlands) or, home-made by spraying the solutions onto standard diet (Transbreed diet from Special Diets Services, UK). During the process of spraying special care was taken to ensure that the solution penetrated the food pellets (the food pellets were rotated and soaked to allow for an even distribution of the solution). The ethanol was allowed to evaporate before supplying the diets to the animals.

For the treatment of adult mice, six-week-old mice were fed the control or the AG-diet for 2 and 4 months. Food intake was monitored by visual inspection of food pellet consumption and body weights were determined twice a week. For the treatment of pre-weaned pups, mating pairs were fed the control or the AG diet from the day of the mating until the pups were 20-days old.

### Cell culture and lipid droplet analyses

Mouse embryonic fibroblasts (MEFs) were isolated from E13.5 WT and Gnpat KO embryos as described [42]. MEFs were cultured in DMEM (Sigma-Aldrich) supplemented with 10% fetal bovine serum and 1x penicillin:streptomycin solution (Gibco). MEFs from WT (n = 4) and Gnpat KO mice (n = 4) at passage 4, were seeded onto glass coverslides in 24-well plates and treated for 7 days with 15 µM of 1-*O*-octadecyl-rac-glycerol. An equal volume of ethanol (vehicle for AG) was added to control-treated cultures of WT and Gnpat KO MEFs.

Lipid droplets in MEFs were visualized after staining with BODIPY 493/503 (Invitrogen). Briefly, cells were fixed with 2% paraformaldehyde for 15 min. After washing in PBS, cells were incubated with BODIPY 493/503 (1∶500 dilution of a 1mg/ml solution) for 15 min. After washing in PBS, cells were mounted on a drop of Vectashield containing DAPI (Vector Labs).

The morphometric analyses of lipid droplets [62] was carried out on z-stacks taken at 63x on a AxioImager Z1 microscope with a 500 µm z-stack step. Images were deconvolved using the quick maximum likelihood estimation (QMLE) algorithm from Huygens Professional (HP) software (Scientific Volume Imaging, Hilversum, The Netherlands). The deconvolved z-stacks were analyzed for number and volume with the object analyzer function of the HP software. These parameters were calculated by the quantifications of all voxels contained in each z-stack and all detected objects were automatically labeled and sent to a continuous Iso Surface Ray tracing renderer. All images shown in Figure 3 are maximum intensity projections (MIP) using the HP renderer option, as described previously [63]. For the untreated cells, 20 photos of each cell line were taken. For control- and AG-treated cells, 40 photos of each cell line were taken.

### Biochemical and western blot analyses

Tissues were homogenized in PBS by sonication. The homogenates were cleared by centrifugation at 900xg for 5 minutes and protein was measured using the DC Protein Assay kit (Bio-Rad) using BSA as standard. Lysates corresponding to 300 µg of protein were used to measure plasmalogens. Plasmalogens were measured as their dimethylacetal derivatives (DMA) by gas chromatography and expressed as the ratio between C18:0 DMA and methylstearate (C18:0) as previously described [64].

White and brown adipose tissues were isolated from WT and Pex7 KO mice (n = 3 per genotype) and lysates were prepared by sonication in PBS containing 0.1% triton X100 and protease inhibitor cocktail (Roche). Protein samples (20ug) were separated on 12.5% SDS-PAGE gels and transferred onto Hybond-C extra membranes (Amersham Biosciences). Membranes were blocked with 10% skim dried milk (Fluka) in PBS containing 0.01% Tween20 and probed with antibodies rabbit anti-UCP1 (Abcam) and mouse anti-β-actin (Sigma-Aldrich). Membranes were developed with ECL after incubation with horseradish peroxidase-labeled secondary antibodies.

### Histological analyses

Pieces of harvested tissues were fixed by immersion in buffered formalin at 4°C for 48 hours, processed for paraffin embedding and sectioned on a Leica RM2255 microtome, according to routine practices. Paraffin sections, 5 µm thick, were deparaffinized in Histoclear II (National Diagnostics), rehydrated using decreasing concentrations of ethanol and used for routine hematoxylin and eosin histological analyses. After fixation, eyes were processed for LR White embedding (Electron Microscopy Sciences) and semi-thin 1 µm sections were cut with a glass knife. Eye sections were stained with Richardson's stain (1∶1 mixture of 1% methylene blue in 1% borax with 1% azure II), dried and mounted with DPX. All sections were analyzed in a Zeiss Axiophot microscope equipped with a Leica DFC320 camera.

For staining lipids, pieces of Harderian gland and brown adipose tissue were fixed in 4% glutaraldehyde in 0.1M sodium cacodylate buffer pH 7.6. Tissues were washed in sodium cacodylate buffer, osmicated, dehydrated and embedded in Embed-812. One µm thin sections were cut on a Leica ultramicrotome, and stained with *p*-phenylenediamine (PPD; 1% solution in methanol). Sections were washed with 95% ethanol, dried and mounted with DPX.

### Electrophysiology

Mice from each genotype and treatment group (n≥3) were anesthetized as described above, and placed on a warm pad at a temperature of 30-34oC. Recordings of compound muscle action potentials (CMAP) were obtained on a PowerLab 4/25T (AD instruments) using Chart5 software [33]. Recording needle electrodes were placed subcutaneously in the foot pad and supramaximal stimulation of sciatic nerves was performed distally at the level of the ankle and proximally at the sciatic notch. Conduction velocities were calculated as: (proximal distance – distal distance)/(proximal latency – distal latency), with latencies corresponding to the time lapse between the stimulus and the onset of the CMAP and expressed in m/s.

### Statistical analysis

All values are expressed as mean ± standard deviation. Statistical comparisons between two experimental groups were evaluated using the Mann Whitney test from the statistical package included in the GraphPad Prism5 software. We considered a *p* value <0.05 as significant.
